# Evaluating drug-drug interaction information in NDF-RT and DrugBank

**DOI:** 10.1186/s13326-015-0018-0

**Published:** 2015-05-11

**Authors:** Lee B Peters, Nathan Bahr, Olivier Bodenreider

**Affiliations:** Lister Hill National Center for Biomedical Communications, National Library of Medicine, National Institutes of Health, Bethesda, Maryland USA; Department of Medical Informatics and Clinical Epidemiology, Oregon Health & Science University, Portland, Oregon USA

**Keywords:** Drug-drug interactions, NDF-RT, DrugBank

## Abstract

**Background:**

There is limited consensus among drug information sources on what constitutes drug-drug interactions (DDIs). We investigate DDI information in two publicly available sources, NDF-RT and DrugBank.

**Methods:**

We acquire drug-drug interactions from NDF-RT and DrugBank, and normalize the drugs to RxNorm. We compare interactions between NDF-RT and DrugBank and evaluate both sources against a reference list of 360 critical interactions. We compare the interactions detected with NDF-RT and DrugBank on a large prescription dataset. Finally, we contrast NDF-RT and DrugBank against a commercial source.

**Results:**

DrugBank drug-drug interaction information has limited overlap with NDF-RT (24-30%). The coverage of the reference set by both sources is about 60%. Applied to a prescription dataset of 35.5M pairs of co-prescribed systemic clinical drugs, NDF-RT would have identified 808,285 interactions, while DrugBank would have identified 1,170,693. Of these, 382,833 are common. The commercial source Multum provides a more systematic coverage (91%) of the reference list.

**Conclusions:**

This investigation confirms the limited overlap of DDI information between NDF-RT and DrugBank. Additional research is required to determine which source is better, if any. Usage of any of these sources in clinical decision systems should disclose these limitations.

**Electronic supplementary material:**

The online version of this article (doi:10.1186/s13326-015-0018-0) contains supplementary material, which is available to authorized users.

## Background

### Motivation

An important component of electronic health record systems is the use of clinical decision support (CDS) to improve medication safety [1,2]. Preventable adverse drug reactions include those resulting from drug-drug interactions (DDIs) [3,4]. Among other things, CDS systems leverage drug-drug interaction information to reduce the possibility of adverse drug events [5,6]. While there are many sources of DDI information, and many commercially available systems which contain this information, there is limited consensus on what constitutes critical and non-critical DDI [7].

One source of publicly available DDI information is contained in the National Drug File Reference Terminology (NDF-RT) and available through the NDF-RT application programming interface (API). Several applications developed for medication management take advantage of the API for checking interactions in medication lists (e.g., the iOS app “Dosage” for medication information and reminders). However, it was announced before the writing of this article that the DDI information would be soon removed from NDF-RT [8]. Our search for another publicly available source of DDI information led us to evaluate the DrugBank data source as a possible replacement for the NDF-RT DDIs.

In this paper, our objective is to evaluate the DDI information in NDF-RT and DrugBank. More specifically, we contrast NDF-RT and DrugBank interactions against each other, and contrast both against a previously published reference set of critical DDIs [9]. We also contrast NDF-RT and DrugBank in their ability to detect DDIs in a large prescription dataset. Finally, we compare the coverage of the reference set by DrugBank and NDF-RT to that of a commercial source.

### Drug information sources

The following sections detail the characteristics of the drug information sources used in this research. We use RxNorm to harmonize drugs between the two sources of DDIs under investigation, NDF-RT and DrugBank.

#### NDF-RT

The National Drug File Reference Terminology (NDF-RT) is a resource developed by the Department of Veterans Affairs (VA) Veterans Health Administration, as an extension of the VA National Drug File [10]. It is updated monthly. NDF-RT covers 7319 active moieties (level = ingredient). In addition to providing information about individual drugs (e.g., mechanism of action, physiologic effect, therapeutic intent), NDF-RT also provides a set of 10,831 drug-drug interactions (DDIs). DDIs are asserted at the ingredient level and accompanied by a mention of severity (significant or critical). For example, NDF-RT asserts a critical interaction between *omeprazole* and *clopidogrel* and asserts a significant interaction between *diltiazem* and *lovastatin*. The version used in this study is dated July 7, 2014 and was accessed through the NDF-RT API [11]. (Provision of DDI information in NDF-RT was discontinued in November 2014).

#### DrugBank

Developed with funding from Genome Canada, DrugBank is a knowledge base containing “extensive biochemical and pharmacological information about drugs, their mechanisms and their targets” [12]. DrugBank covers 7683 active moieties. Although not primarily developed for clinical use, DrugBank provides a set of 12,128 drug-drug interactions (DDIs), asserted at the ingredient level, along with a brief textual description of the interaction, and information about the possible molecular basis of the interaction (target-based, enzyme-based, transporter-based). For example, DrugBank asserts an interaction between *omeprazole* and *clopidogrel bisulfate*, described as “Omeprazole may decrease serum concentrations of the active metabolite(s) of clopidogrel. Clopidogrel prescribing information recommends avoiding concurrent use with omeprazole, due to the possibility that combined use may result in decreased clopidogrel effectiveness”. The possible molecular basis of the interaction is reported to be enzyme-based or transporter-based. The version used in this study (4.0) was downloaded from the DrugBank website on July 1, 2014.

#### RxNorm

RxNorm is a standardized nomenclature for medications produced and maintained by the U.S. National Library of Medicine (NLM) [13]. While NDF-RT is one of the drug information sources integrated in RxNorm, DrugBank is not. However, most ingredients from DrugBank are covered by RxNorm and RxNorm can be used to map drugs between DrugBank and NDF-RT. Moreover, RxNorm provides a rich network of relations among various types of drug entities. For example, RxNorm explicitly asserts that *clopidogrel bisulfate* is the “precise ingredient” of the ingredient *clopidogrel*, making it possible to normalize the various salts and esters of a drug to their base form. RxNorm also integrates the Anatomical Therapeutic Chemical (ATC) classification, which makes it possible to extract the ATC class for most drugs. For example ATC classifies *clopidogrel* as a *Platelet aggregation inhibitors excl. heparin*. The July 2014 version of RxNorm is used in this study and was accessed through the RxNorm API [14].

### Related work

Extracting drug-drug interaction (DDI) information from textual resources, such as the biomedical literature or structured product labels is an active field of research [15-18]. Other researchers predict DDIs from a variety of resources [19].

As mentioned earlier, researchers have shown the benefit of integrating DDI information in CPOEs (e.g., [1,2]). However, not all studies demonstrate improvement on medication safety, especially due to the large number of alerts produced by some systems [20], which raises questions about the quality of the underlying DDI information.

Given the limited consensus across sources of DDI information [7], researchers have proposed criteria for assessing high-priority DDIs [21] and for calibrating CDS systems [22]. An expert panel was convened to identify high-severity, clinically significant DDIs for the Office of the National Coordinator for Health Information Technology (ONC) as part of the *Meaningful Use* incentive program [9]. Candidate DDIs were assessed by the panel based on a number of factors, including severity levels across medication knowledge bases, consequences of the interaction, predisposing factors, and availability of therapeutic alternatives. The resulting list contains 360 interacting pairs of individual drugs containing 86 unique drugs. This list will be referred to as the reference set of DDIs in the following sections.

### Specific contribution

The specific contribution of our work is to contrast two publicly available sources of DDI information, NDF-RT and DrugBank, through an assessment of the overlap of their content, and of their coverage of a reference set of DDIs. Moreover, we compare the ability of these two sources to identify DDIs in a large prescription dataset, and contrast them against a commercial source. To the best of our knowledge, this is the first such comparative investigation of NDF-RT and DrugBank DDI information.

## Methods

Our approach to evaluating drug-drug interaction (DDI) information in NDF-RT and DrugBank can be summarized as follows. We acquire the list of drug-drug interactions in NDF-RT and DrugBank, as well as a reference set of DDIs. We map all drugs from the three sets to RxNorm and further normalize them to ingredient entities. We then compare the lists of pairs of interacting drugs across sources in order to determine the shared coverage between NDF-RT and DrugBank, as well as the coverage of the reference set by both sources. We characterize the differences among DDI sets in terms of drug classes. We also compare the interactions detected with NDF-RT and DrugBank in a large prescription dataset. Finally, we compare the coverage of the reference set by DrugBank and NDF-RT to that of a commercial source.

### Acquiring DDI information

#### NDF-RT

We used the NDF-RT API [11] to first extract the full set of DDIs (DRUG_INTERACTION_KIND concepts), then to extract each associated drug concept (level = ingredient) in the pair.

#### DrugBank

The DrugBank XML and schema definition files were downloaded from the DrugBank web site. We extracted the interaction data from the XML file and created a table of drug name pairs for the interacting drugs.

#### Reference set

The reference set of DDIs was created from the drug names listed in Table two of [9] by associating each object drug with all corresponding precipitant drug(s) within a given interaction class. One pair involving a multi-ingredient drug (*azathioprine and mercaptopurine*) was eliminated, because multi-ingredient drugs are generally not consistently represented across sources.

### Normalizing drugs in reference to RxNorm

After obtaining the drug name pairs for interacting drugs, we mapped the drugs to RxNorm by retrieving the RxNorm identifiers (RxCUIs). For NDF-RT, the RxCUI is part of the drug’s concept properties, so we used the NDF-RT API to extract the RxCUI from the drug properties. For DrugBank and the reference set, we used the RxNorm API [7] to find the RxCUI from the drug name. More specifically, we used exact and normalized string matches to map drug names to RxNorm.

We then “normalized” to RxNorm ingredients those drugs which mapped to RxNorm entities. Some of the drugs corresponded to base ingredients (e.g., *doxacurium*), while others corresponded to salt forms that RxNorm classifies as “precise ingredients” (e.g., *doxacurium chloride*). In order to establish a consistent drug representation across all three data sets for comparison, we converted precise ingredients to RxNorm ingredients (e.g., *doxacurium chloride* to *doxacurium*) using the RxNorm API.

We eliminated from the comparison those pairs for which at least one of the drugs could not be found in RxNorm. For example, the DrugBank drug *cerivastatin*, which was withdrawn from the U.S. market in 2001, is not present in RxNorm.

### Comparing interactions across sources

Having normalized all drugs to RxNorm ingredients, we compared the lists from DDIs of NDF-RT and DrugBank in order to determine the similarities and unique features of each source. In addition, we compared each with the reference set of DDIs to determine how many of the reference DDIs each source covered.

### Characterizing differences

To determine if there was a pattern of missing interactions, we abstracted the pairs of interacting drugs into pairs of classes from the Anatomical Therapeutic Chemical (ATC) Classification System. We mapped the drugs directly to their 4^th^-level ATC classes using the RxNorm API and identified those pairs of ATC classes for which the proportion of DDIs in common between NDF-RT and DrugBank was low, using the Jaccard score. Of note, some NDF-RT and DrugBank drugs are not represented in ATC. So only DDIs for which both the object and precipitant drugs are present in ATC were analysed.

### Comparing coverage of interactions from actual prescription data

In order to assess the difference between NDF-RT and DrugBank interactions based on usage data, we collected drug pairs generated from actual patient prescription lists. Checking medication lists for drug-drug interactions represents a practical use case and is offered as a service by many medication list applications. It also provides some frequency of usage of the co-prescribed drug pairs extracted from the lists. The data was acquired from Symphony Health Solutions (http://symphonyhealth.com/). It included one year of prescription data from the Washington, D.C. area from July 1, 2011 through June 30, 2012. Prescription information included prescriber, de-identified patient information, and specific medication information, including the drug name and strength. The drug information was mapped into RxNorm clinical drugs. From a list of co-prescribed drugs, we generated all possible pairwise combinations within the drug list (ignoring the order of drugs in the pair, since the distinction between object and precipitant drugs is not required for DDI testing). We ignored topical drugs from the prescription list, because DDI information represented at the ingredient level in NDF-RT and DrugBank usually refers to interactions between systemic drugs. For example, we generated the following pair of RxNorm clinical drug, *24 HR Diltiazem Hydrocloride 360 MG Extended Release Oral Capsule (830795)* and *Lovastatin 20 MG Oral Tablet (197904)*. We mapped the clinical drugs to their active moieties in RxNorm (*Diltiazem Hydrocloride* and *Lovastatin*, respectively) and further normalized those to RxNorm ingredients, as we did with all object and precipitant drugs from the various DDI lists. Here, we normalized *Diltiazem Hydrocloride* to *Diltiazem*. In summary, we transformed the pair of RxNorm clinical drugs extracted from the prescription list into the pair of RxNorm ingredients (*Diltiazem*, *Lovastatin*) for comparison to the DDIs in NDF-RT and DrugBank.

### Publicly available vs. commercial DDI sources

To compare the coverage of the reference set by DrugBank and NDF-RT to that of a commercial source, we investigated the Multum drug knowledge base through the interaction checker of the website Drugs.com (http://www.drugs.com/), against which we tested the 360 DDIs of the reference set.

## Results

### DDI information in NDF-RT, DrugBank and the reference set

Table 1 summarizes the number of DDIs in the three data sets.

**Table 1:** **DDI counts in the three datasets**

**Data set**	**DDI counts**		
	***Total from source***	***Mapped to RxNorm***	***Normalized***
Reference	360	360	360
DrugBank	12128	11762	11552
NDF-RT	10831	9452	9392

#### Reference set

The reference set contained 360 DDIs; all the drugs mapped to RxNorm and were all classified as ingredients. The 360 DDIs covered 86 RxNorm ingredients.

#### DrugBank

DrugBank contained 12,128 DDIs defined in the XML file. DDIs involving drugs with no mapping to RxNorm were discarded (418 DDIs involving 46 drugs). Analysis of all the discarded DDIs revealed several reasons why the DDIs were eliminated. Some DDIs involved drugs that were either withdrawn from public use (e.g., *cerivastatin, ephedra, heptabarbital*) or not approved by the U.S. Food and Drug Adminstration (e.g., *cinolazepam, carbetocin*) and those drugs could not be mapped to RxNorm. Additionally, 518 DrugBank DDIs were eliminated through the ingredient normalization process. For example, the DDIs containing *zuclopenthixol, zuclopenthixol acetate* and *zuclopenthixol deconoate* were normalized to produce a single set with *zuclopenthixol* as the ingredient, eliminating the redundant pairs containing the salt forms. The resulting 11,552 normalized DDIs covered 1153 RxNorm ingredients.

#### NDF-RT

NDF-RT contained 10,831 DDIs extracted from the data set. DDIs involving drugs with no mapping to RxNorm were discarded (1379 DDIs involving 38 drugs). Analysis of all the discarded DDIs revealed that some DDIs were associated with drugs which referenced obsolete RxNorm concepts, many of these vaccine drugs that were recently removed from RxNorm. Additionally, 60 NDF-RT DDIs were eliminated through the ingredient normalization process. The resulting 9,392 normalized DDIs covered 1079 RxNorm ingredients.

In the remainder of this paper, DDIs refer to pairs of object and precipitant drugs normalized to RxNorm ingredients. However, even after normalization to RxNorm ingredients, the coverage of drugs is not expected to be the same in NDF-RT and DrugBank. For example, vaccines and other biologicals are present in NDF-RT, but out of scope for DrugBank. When analysing DDIs across the two sources, breakdown by pharmacological classes will reflect such differences in drug coverage.

### Comparing interactions across sources

The matching DDIs between the three data sets are shown in Table 2.

**Table 2:** **Matching DDIs across data sets**

**Data set**	**Number of matching DDIs**		
	***Reference***	***DrugBank***	***NDF-RT***
Reference	360	211	207
DrugBank	211	11552	2801
NDF-RT	207	2801	9392

#### Overlap between DrugBank and NDF-RT

Overall, the 2801 DDIs common to NDF-RT and DrugBank represent a 30% coverage rate of NDF-RT by DrugBank and a 24% coverage rate of DrugBank by NDF-RT. Example of common DDIs include *diltiazem/lovastatin* and *itraconazole/sirolimus*. Examples of DDIs in DrugBank only include *acebutolol/Insulin Lispro* and *metronidazole/terfenadine*. Examples of DDIs in NDF-RT only include *amiodarone/sotalol* and *meperidine/linezolid*.

When we only consider DDIs from the reference set, the overlap between DrugBank and NDF-RT is significantly higher. The 146 reference DDIs common to NDF-RT and DrugBank represent 71% of the 207 reference DDIs covered by NDF-RT and 69% of the 211 reference DDIs covered by DrugBank.

#### Reference set coverage

Table 3 shows the breakdown by the reference set groups of the DDI mapping for DrugBank and NDF-RT. DrugBank contained 211 DDIs (59%) from the reference set, compared with 207 DDIs (58%) for NDF-RT. There were 146 DDIs (42%) from the reference set which were both in the NDF-RT and DrugBank, including *diltiazem/lovastatin, simvastatin/amiodarone* and *atazanavir/omeprazole*. Conversely, there were 88 DDIs (24%) in the reference set which were not contained in either DrugBank or NDF-RT, including *irinotecan/indinavir, ketoconazole/ergonovine* and *milnacipran/selegiline*. DrugBank contained 65 DDIs from the reference set which were not in NDF-RT (e.g., *lovastatin/tipranavir* and *ramelteon/fluvoxamine*), and NDF-RT contained 61 DDIs from the reference set not in DrugBank (e.g., *fluoxetine/procarbazine* and *simvastatin/saquinavir*). DrugBank had coverage in all groups from the reference set, though only 100% coverage in two groups. NDF-RT had coverage of all but one group (#22 *ramelteon-fluvoxamine*). The coverage of the reference set of drug-drug interactions by DrugBank and NDF-RT is available as Additional file 1.

**Table 3:** **Reference Set DDI and coverage in NDF-RT and DrugBank**

**Grp #**	**DDI group description**	**# pairs**	**NDF-RT matches**	**DrugBank matches**	**Object members**	**Precipitant members**
3	Amphetamine and derivatives – MAO inhibitors	60	30 (50%)	30 (50%)	Dexmethylphenidate, Dextroamphetamine, Methylphenidate, Lisdexamfetamine, Phendimetrazine, Pseudoephedrine, Amphetamine, Benzphetamine, Diethylproprion, Phentermine, Atomoxetine, Methamphetamine	Tranylcypromine, Phenelzine, Isocarboxazid, Procarbazine, Selegiline
4	Atazanavir – Proton pump inhibitors (PPIs)	5	5 (100%)	5 (100%)	Atazanavir	Omeprazole, Lansoprazole, Pantoprazole, Rabeprazole, Esmoprazole
8	Fluoxetine - MOA inhibitors	55	39 (71%)	43 (78%)	Fluoxetine, Paroxetine, Citalopram, Escitalopram, Sertraline, Fluvoxamine, Duloxetine, Nefazodone, Desvenlafaxine, Milnacipran, Venlafaxine	Tranylcypromine, Phenelzine, Isocarboxazid, Procarbazine, Selegiline
11	Irinotecan – Ketoconazole	23	1 (4%)	5 (22%)	Irinotecan	Ritonavir, Nelfinavir, Atazanavir, Indinavir, Saquinavir, Amprenavir, Darunavir, Lopinavir, Tipranavir, Fosamprenavir, Clarithromycin, Erythromycin, Telithromycin, Amiodarone, Verapamil, Diltiazem, Ketoconazole, Itraconazole, Fluconazole, Voriconazole, Nefazodone, Aprepitant, Cimetidine
16	Narcotic analgesics – MAO inhibitors	30	25 (83%)	15 (50%)	Meperidine, Methadone, Tapentadol, Fentanyl, Tramadol, Dextromethorphan	Tranylcypromine, Phenelzine, Isocarboxazid, Procarbazine, Selegiline
22	Ramelteon-fluvoxamine	4	0 (0%)	2 (50%)	Ramelteon	Fluvoxamine, Amiodarone, Ticlopidine, Ciprofloxacin
23	Rifampin – ritonavir	60	41 (68%)	25 (42%)	Bosentan, Rifapentine, Carbamazepine, Rifabutin, Rifampin, St. John’s wort	Ritonavir, Nelfinavir, Atazanavir, Indinavir, Saquinavir, Amprenavir, Darunavir, Lopinavir, Tipranavir, Fosamprenavir
25	HMG Co-A reductase inhibitors – protease inhibitors	40	38 (95%)	33 (83%)	Simvastatin, Lovastatin	Ritonavir, Nelfinavir, Atazanavir, Indinavir, Saquinavir, Amprenavir, Darunavir, Lopinavir, Tipranavir, Clarithromycin, Erythromycin, Telithromycin, Amiodarone, Verapamil, Diltiazem, Tranylcypromine, Phenelzine, Isocarboxazid, Procarbazine, Selegiline
27	Telithromycin – ergot alkaloids and derivatives	60	13 (22%)	35 (58%)	Ritonavir, Nelfinavir, Atazanavir, Indinavir, Saquinavir, Amprenavir, Darunavir, Lopinavir, Tipranavir, Clarithromycin, Erythromycin, Telithromycin, Ketoconazole, Itraconazole, Voriconazole	Ergotamine, Methylergonovine, Dihydroergotamine, Ergonovine
28	Tizanidine – ciprofloxacin	7	6 (86%)	6 (86%)	Tizanidine	Fluvoxamine, Amiodarone, Ticlopidine, Ciprofloxacin, Mexiletine, Propafenone, Zileuton
30	Tranylcypromine – procarbazine	1	1 (100%)	1 (100%)	Tranylcypromine	Procarbazine
31	Triptans – MAO inhibitors	15	8 (53%)	11 (73%)	Sumatriptan, Zolmitriptan, Rizatriptan	Tranylcypromine, Phenelzine, Isocarboxazid, Moclobamide, Methylene blue
	**TOTAL**		**207 (58%)**	**211 (59%)**		

Of the 86 drugs contained in the reference set, one (*moclobamide*, involved in 3 DDIs) did not exist in NDF-RT and two (*dexmethylphenidate*, involved in 5 DDIs and *methylene blue*, involved in 3 DDIs) did not exist in DrugBank. In addition, there were four other drugs *(bosentan, lopinavir, methadone* and *zileuton*) which were present in DrugBank, but not involved in any of the DDIs from the reference set. DrugBank did have DDIs for these four drugs outside of the reference set.

#### Pharmacologic classes

We mapped the DDIs from NDF-RT and DrugBank to 4^th^-level ATC drug classes to see if there were distinctive class differences between the two sets of DDIs. A small proportion of the drugs, such as *avanafil*, *lopinavir* and *zileuton* (14% in NDF-RT, 11% in DrugBank) were not represented in ATC and the corresponding DDIs were excluded from the analysis.

Table 4 shows the top frequency count of ATC classes for NDF-RT and DrugBank DDIs, along with the number of drugs from each class represented in the source of DDI. For example, NDF-RT has 401 DDIs involving 11 drugs for the *fluoroquinolone* class, while DrugBank has 335 interactions involving 14 drugs for this class. *Fluoroquinolones* is in the top-10 classes for the frequency of DDIs in NDF-RT, but not in DrugBank. Two classes, *protein kinase inhibitors* and *selective immunosuppressants*, seem underrepresented in DrugBank, while three classes, *anticholinesterases*, *hydantoin derivatives* and *non-selective monoamine reuptake inhibitors*, seem underrepresented in NDF-RT.

**Table 4:** **Top ATC Class Counts in NDF-RT and DrugBank**

	**NDF-RT**		**DrugBank**	
**ATC Class Name**	***DDI***	***drugs***	***DDI***	***drugs***
Antibiotics	**709**	22	**744**	25
Anticholinesterases	25	4	**411**	7
Fluoroquinolones	**401**	11	335	14
Hydantoin derivatives	266	4	**406**	4
Macrolides	**443**	6	**502**	9
Non-selective monoamine reuptake inhibitors	**311**	10	**736**	10
Other antidepressants	**309**	11	**457**	10
Protease inhibitors	**1130**	11	**751**	11
Protein kinase inhibitors	**759**	19	**389**	20
Selective immunosuppressants	**307**	13	150	14
Selective serotonin reuptake inhibitors	**341**	6	380	6
Triazole derivatives	**427**	4	**502**	4
Vitamin K antagonists	292	3	**427**	3

*Boldface values indicate the ten top categories in each source.

Table 5 shows the ATC pairs containing the most DDIs from NDF-RT and DrugBank, but a low proportion of shared DDIs between the two sources (evidenced by the low Jaccard scores). The *Adrenergic and dopaminergic agents - Non-selective monoamine reuptake inhibitors* class pair, for example, has no NDF-RT DDIs and 78 DrugBank DDIs. Examples in this class pair include *dopamine/amitriptyline* and *norepinephrine/amoxipine*. Conversely, in the *Protease inhibitors - Protein kinase inhibitors* class pair, many more specific DDIs exist in NDF-RT, such as *boceprevir/bosutinib*, than in DrugBank. Finally, the *Beta blocking agents, non-selective - Sulfonamides, urea derivatives* class pair illustrates a situation where both sources share a majority of DDIs, but also have a significant number of specific DDIs. For example, *sotalol/glyburide* is common, but *sotalol/tolazamide* is specific to NDF-RT and *sotalol/gliclazide* is specific to DrugBank.

**Table 5:** **Top ATC Class Pairs**

**ATC Class Pair**	**total DDIs**	**NDF-RT only**	**DrugBank only**	**both**	**Jaccard**
Protease inhibitors - Protein kinase inhibitors	103	72	10	21	0.20
Benzodiazepine derivatives - Protease inhibitors	88	5	52	28	0.32
Adrenergic and dopaminergic agents - Non-selective monoamine reuptake inhibitors	78	0	78	0	0
Antibiotics - Other quaternary ammonium compounds	71	35	8	28	0.39
Penicillins with extended spectrum - Tetracyclines	63	0	58	5	0.08
Protein kinase inhibitors - Triazole derivatives	60	32	10	18	0.30
Beta blocking agents, non-selective - Sulfonamides, urea derivatives	56	10	14	32	0.57
Macrolides - Protein kinase inhibitors	56	27	14	15	0.27

#### Comparing coverage of interactions from actual prescription data

**Figure 1:**
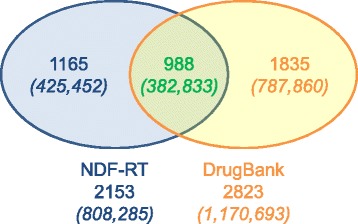
Frequency of DDIs in prescription pairs.

From the prescription dataset, almost 35.9 million pairs were extracted, representing 816,258 unique pairs of RxNorm clinical drugs. Restricted to systemic drugs, the dataset included 35.5 million pairs of clinical drugs (808,285 unique). Each clinical drug maps to at least one ingredient, and multi-ingredient drugs map to several ingredients, resulting in multiple ingredient pairs for a given pair of clinical drugs. For example, starting from the pair of clinical drugs *Primidone 250 MG Oral Tablet* and *Carbidopa 25 MG/Levodopa 100 MG Oral Tablet*, we generate the following two pairs of ingredients, (*Primidone*, *Carbidopa*) and (*Primidone*, *Levodopa*), because the clinical drug *Carbidopa 25 MG/Levodopa 100 MG Oral Tablet* maps to two ingredients, *Carbidopa* and *Levodopa*. After mapping of the clinical drugs to ingredients, there were 45.2 million pairs of co-prescribed drugs, ranging in frequency between 1 and 158,515 (median = 18). Of these, 808,285 pairs matched with NDF-RT DDIs (2153 unique), while 1,170,693 pairs matched with DrugBank DDIs (2823 unique). There were 382,833 pairs that matched with both NDF-RT and DrugBank (988 unique). There were 26,672 matches with the reference set (88 unique). Figure 1 shows the frequency of the DDIs (in parenthesis) with the unique number of DDIs found in the prescription dataset.

Examples of frequently co-prescribed drugs identified as a DDI by both NDF-RT and DrugBank include *Diltiazem*/*Lovastatin* and *Clarithromycin*/*Simvastatin*. Co-prescribed drugs identified as a DDI by NDF-RT, but not DrugBank include *Amlodipine*/*Simvastatin* and *Diltiazem*/*Oxycodone*. Conversely, DDIs identified by DrugBank, but not NDF-RT, include *Amoxicillin*/*Ethinyl Estradiol* and *Hydrochlorothiazide*/*Insulin Lispro*. Table 6 shows the most frequently co-prescribed pairs recognized as a DDI by one or both of the sources.

**Table 6:** **Top DDI Pairs found in Prescription Data**

**Drug pair**	**Freq**	**DrugBank DDI**	**NDF-RT DDI**
Amlodipine-Simvastatin	76980		X
Glipizide-Metoprolol	15469	X	X
atorvastatin-Fenofibrate	14897	X	X
Fenofibrate-Simvastatin	14060	X	X
Albuterol-Metoprolol	14030	X	
Insulin Glargine-Metoprolol	13492	X	
Metoprolol-Sertraline	13311	X	
atorvastatin-Diltiazem	13218	X	X
Digoxin-Furosemide	12958	X	
Lisinopril-Triamterene	12399	X	X
cyclobenzaprine-Tramadol	12211	X	
Diltiazem-Metoprolol	11738	X	
Oxycodone-Sertraline	11710	X	
Diltiazem-Simvastatin	11484	X	X
Alprazolam-Fluoxetine	11194		X
Sertraline-Trazodone	11066	X	
Amoxicillin-Ethinyl Estradiol	11044	X	
Lisinopril-Spironolactone	10248	X	X
Clonazepam-Fluoxetine	10149		X
Fenofibrate-rosuvastatin	9954	X	X
Clonidine-Metoprolol	9827	X	X
Hydralazine-Metoprolol	9556	X	
duloxetine-Trazodone	9236	X	
clopidogrel-pantoprazole	9177	X	
Escitalopram-Metoprolol	8396	X	
Levofloxacin-Prednisone	8335		X
Escitalopram-Oxycodone	8228	X	
Citalopram-Trazodone	8081	X	
Citalopram-Oxycodone	8016	X	
Metoprolol-salmeterol	7703	X	
Atenolol-Glipizide	7597	X	X
Fluoxetine-Trazodone	7579	X	
carvedilol-Digoxin	7517	X	
Fluoxetine-Oxycodone	7508	X	
carvedilol-Insulin Glargine	7496	X	
Citalopram-Metoprolol	7474	X	
Escitalopram-Trazodone	7421	X	
Alprazolam-Omeprazole	7339	X	

#### Publicly available vs. commercial DDI sources

We were able to match 328 (91%) DDIs of the reference set to Multum DDIs, a significantly higher proportion compared to NDF-RT and DrugBank. The level of severity provided by Multum for these DDIs (on a 3-level scale *mild, moderate* or *major*) was consistent with the notion of “high-severity” claimed by the authors of the reference set, since 302 DDIs were rated major and the other 26 were rated moderate.

## Discussion

In this section, we discuss some of the reasons for the limited overlap of DDI information among sources. We also analyse its implications for our interactions API.

### Limited overlap between NDF-RT and DrugBank DDIs

#### Findings

One important finding of this investigation is the limited overlap (<30%) between DrugBank and NDF-RT DDIs, although both sources cover roughly the same number of DDIs. We found 6591 DDIs specific to NDF-RT and 8751 specific to DrugBank, while only 2801 DDIs were common to both sources. Differences in scope (e.g., the lack of vaccine DDIs in DrugBank) account for only a small portion of the differences. Differential coverage persisted after abstracting DDIs to the corresponding drug classes from ATC. For example, NDF-RT had many more DDIs than DrugBank for *Protease inhibitor* drugs, and had much fewer DDIs for *Anticholinesterases* drugs. Likewise, many ATC class pairs had a small proportion of DDIs in common, evidenced by low Jaccard scores. For example, among the 56 DDIs for the *Macrolides - Protein kinase inhibitors* class pair, only 15 are common to NDF-RT and DrugBank (Jaccard = 0.27). The low overlap rate also applied to our more frequently prescribed drug pairs in the prescription data set. When we examined the co-prescribed drugs from the prescription data set, we found significant differences in identification of DDIs between the two sources for the most frequently co-prescribed pairs. Of the top 10 most frequently prescribed drug pairs identified as potential DDIs by DrugBank, only 5 were recognized as NDF-RT DDIs (see Table 6 for details).

#### DDI severity

We wondered if the differential coverage observed between NDF-RT and DrugBank could be due in part to different editorial guidelines for curating less severe DDIs, assuming the most severe DDIs would be covered more consistently by both sources. This did not seem to be the case since the 360 high-severity DDIs from the reference set are only partially covered by NDF-RT and DrugBank. While NDF-RT provides an indication of severity (*critical* or *significant*) for its DDIs, DrugBank does not. Nevertheless, we tested if DrugBank would cover a larger proportion of NDF-RT *critical* DDIs. In fact, DrugBank DDIs accounted for 31% of the total *critical* DDIs in NDF-RT and for 29% of the total *significant* DDIs. In other words, there is no difference in the coverage of NDF-RT by DrugBank in terms of DDI severity. (Even when correcting for the absence of vaccine DDIs in DrugBank, the overlapping DDIs accounted for just 40% of the *critical* DDIs in NDF-RT.)

#### Differential coverage of DDIs among sources

Our finding of limited overlap between NDF-RT and DrugBank should not be surprising given similar findings in studies comparing drug knowledge bases for critically important DDIs [21]. There is no standardization for what constitutes a drug-drug interaction, and DDI curators have to consider a variety of drug and patient specific factors in their decision to include pairs of drugs in their DDI lists. These factors include the severity of the interaction, the probability of the interaction, patient characteristics (e.g., specific patient groups, such as elderly patients), and evidence supporting the interaction (quantity of evidence, as well as the quality of the evidence). The number of factors to consider makes this a complex and daunting task. Moreover, the maintenance of DDI lists itself is an issue, as additional evidence for or against DDIs becomes available over time. Finally, different strategies for prioritizing the various factors also accounts for some of the differences observed across sources.

### Implications for the interactions API

Overall, our investigation of NDF-RT and DrugBank as sources of DDIs for our API provides a mixed picture. Not only do they both provide incomplete coverage of the reference set (about 60% each), but their overlap is also limited (42%). A similar difference could be observed when we simulated interaction detection based on actual usage data. We also confirmed that a commercial source, Multum, provided a more systematic coverage of the reference set.

With the provision of DDI information being discontinued in NDF-RT in November 2014, we decided to use DrugBank as a replacement for our interactions API. This solution is far from ideal, because this investigation did not establish the accuracy of either source, but simply assessed differences among them. On the other hand, DDI determination is not an exact science and both NDF-RT and DrugBank provide valuable information to support medical decision. NDF-RT DDIs are associated with levels of severity, while DrugBank’s come with a short description. In practice, the absence of severity levels in DrugBank is a disadvantage, as severity is an important consideration for determining when to alert physicians to a potential DDI. Severity is also a requirement when checking DDI in the context of the *Meaningful Use* incentive program.

It is important to keep in mind that no clinical decision system, as good as it is, can be a substitute for medical advice. Our API not only clearly discloses the origin of the information it provides, but also reminds our users to seek advice from health professionals before making decisions about their medications. In the future, we plan to perform a more systematic and qualitative investigation of publicly available and commercial DDI sources in order to better assess the differences among these sources.

## Conclusions

This study is the first comparative investigation of DDI information in two publicly available sources, NDF-RT and DrugBank. We compared the two sources not only to themselves, but also to a reference set of DDIs, and assessed their ability to identify DDIs in a large prescription dataset. We also contrasted NDF-RT and DrugBank against the commercial source Multum.

This investigation confirms the limited overlap between DDI information between NDF-RT and DrugBank, even for the reference dataset. Additional research is required to determine which source is better, if any. Usage of any of these sources in clinical decision systems should clearly disclose these limitations.

## Additional file

Additional file 1:
**Coverage of the reference set of drug-drug interactions by DrugBank and NDF-RT.**
